# Assessing the status of sundial lupine (*Lupinus perennis* L.) genetic diversity and population structure throughout its distribution

**DOI:** 10.1093/aobpla/plaf047

**Published:** 2025-09-02

**Authors:** Isabella R Petitta, Autumn E Sabo, Margarita M López-Uribe

**Affiliations:** Intercollege Graduate Degree Program in Ecology, The Pennsylvania State University, University Park, PA, United States; Department of Entomology, The Pennsylvania State University, University Park, PA, United States; Intercollege Graduate Degree Program in Ecology, The Pennsylvania State University, University Park, PA, United States; Biology Program, The Pennsylvania State University, Monaca, PA, United States; Intercollege Graduate Degree Program in Ecology, The Pennsylvania State University, University Park, PA, United States; Department of Entomology, The Pennsylvania State University, University Park, PA, United States; Evolution & Diversity

**Keywords:** conservation, inbreeding, fragmentation, microsatellite

## Abstract

Habitat loss and fragmentation directly influence plant genetic diversity and how it is spatially structured. Species associated with shrinking habitats generally experience population declines and genetic erosion, potentially increasing extinction risk. An endangered North American habitat, the oak savanna, supports high plant biodiversity and is the primary habitat for sundial lupine (*Lupinus perennis* L.). This legume serves as the primary host plant for several highly specialized insects. Sundial lupine is declining in the eastern part of its range, and restoration efforts lack an understanding of the regional level of population differentiation in this species. In this study, we addressed this gap by characterizing the population genetic structure and levels of inbreeding of sundial lupine with nine microsatellite markers across 25 populations throughout its distribution. To assess whether losses of genetic diversity are impacting population fitness, we investigated whether germination rates were associated with within-population genetic diversity and inbreeding. Genetic diversity was greatest in the southernmost population (Florida). We found significant differentiation between populations (pairwise *G*_ST_ ranging from 0.003 to 0.63) and identified five genetic clusters with high levels of admixture. No sites showed significant levels of inbreeding (*F*_IS_; mean −0.01, standard deviation 0.15). Germination success did not differ based on population size but decreased in populations with negative inbreeding coefficients. Our data suggest that there are significant levels of admixture between populations; thus, it is possible to use seeds from multiple sources for restoration. Still, due to the widespread distribution of sundial lupine, it is possible that populations may exhibit local adaptation to regional aspects of their habitat, and we caution against long-distance movement between populations.

## Teaser

Habitat loss threatens plant genetic diversity, with consequences for species survival and ecosystem health. This study describes the genetic diversity, structure, and inbreeding of Sundial lupine (Lupinus perennis L.), a declining legume critical to dependent insect species. We identified five distinct genetic clusters and substantial population differentiation with the use of nine microsatellite markers and 25 populations. We report that no populations have significant inbreeding, but lower germination was linked to negative inbreeding coefficients. These findings highlight genetically unique populations, offering guidance for effective restoration and conservation of this ecologically relevant species.

## Introduction

Habitat loss is the primary threat to global biodiversity and the main driver of declines in plants of conservation concern (Giam et al. 2010). Declining plant species typically have limited dispersal abilities and high habitat specificity, restricting their capacity to colonize areas beyond their primary habitat (Slatyer et al. 2013, Vincent et al. 2020). Reductions in population size also influence the persistence of declining plant species through decreased genetic diversity, which can further reduce growth and reproduction (Honnay and Jacquemyn 2007, Aguilar et al. 2008). Additionally, low levels of genetic diversity can hinder species’ potential to adapt, decreasing resilience to environmental change (Chung et al. 2023).

Certain life history traits such as mating system, life-span, and rarity influence plant genetics (Aguilar et al. 2008, González et al. 2020). Among these, the mating system—whether selfing, mixed, or outcrossing—stands out as a key determinant of genetic diversity in plants (He et al. 2024). Several empirical studies support the expectation that self-compatible plant taxa show lower neutral genetic diversity compared with other mating systems (Glémin et al. 2006, Guo et al. 2009, Koelling et al. 2011). Outcrossing plant species generally exhibit higher genetic variation within populations compared with selfing plant species; thus, reductions in effective population size of outcrossing species may have stronger negative effects on genetic diversity and fitness (Hamrick and Godt 1997). Outcrossing plants depend on pollen dispersal agents, such as animals or wind, to ensure successful pollen transfer and promote gene flow (Cresswell 2006). In small populations, reductions in available floral display area can reduce pollinator attraction and pollen dispersal rates, which are critical for outcrossing (Bauer et al. 2017, Erickson et al. 2022). Thus, the negative impacts of reductions in population size are more severe in outcrossing species that rely on pollinators for reproduction and can experience inbreeding depression from selfing or mating with genetically related individuals.

For plant populations that persist in endangered habitats or have restricted distributions, evaluating population genetics is important for several reasons. First, it helps the identification of populations with low genetic diversity and high levels of inbreeding that may be at risk of local extinction. Secondly, assessing genetic diversity facilitates the identification of genetically unique populations that may require targeted conservation efforts or could serve as sources for restoring small, declining populations. A review analysing studies on the management of small, fragmented populations found that 56% did not discuss genetic risks, such as inbreeding and outbreeding depression, when making recommendations for management (Liddell et al. 2021). Restoration efforts without knowledge of the levels and distribution of genetic diversity can potentially cause losses of unique genetic lineages through admixture or outbreeding depression from the mating of genetically distant individuals that are adapted to different local environments (Fischer and Matthies 1997, Montalvo and Ellstrand 2001, Aitken and Whitlock 2013). Therefore, population genetic data are essential for making evidence-based conservation decisions and should be considered to enhance the preservation of rare plants.

Globally, temperate savannas support rich plant diversity with an average of 16.1 species per square metre but are one of the least protected biomes (Leach and Givnish 1999, Hoekstra et al. 2005). In North America, oak savannas currently cover <0.02% of their original land area (Nuzzo 1986, Hoekstra et al. 2005). Alarmingly, their decline jeopardizes the diverse plant taxa they sustain, including trees, shrubs, ferns, graminoids, and forbs (Abella et al. 2004). Some savanna-associated plant species are considered of conservation concern, including purple milkweed (*Asclepias purpurascens* L.), yellow giant hyssop (*Agastache nepetoides* (L.) Kuntze), and sundial lupine (*Lupinus perennis* L.; Leach and Givnish 1999, Toomey and Toomey 2002, Brock 2009, Petitta et al. 2023). Despite declines, limited population genetic studies have focused on oak savanna–associated plants and have predominantly focused on the population genetics of oak species (Craft and Ashley 2007, Kittelson et al. 2009).

Sundial lupine serves as the larval food source for three butterflies of conservation concern, including the federally endangered Karner blue butterfly (*Plebejus samuelis* (Nabokov, 1944); Swengel and Swengel 1996). Throughout its range in North America, sundial lupine is a species of concern in over 50% of its distribution due to primary habitat loss (Petitta et al. 2023). Populations of sundial lupine in the midwestern United States, such as Michigan (USA), Ohio (USA), and Wisconsin (USA), persist in managed oak savannas, oak barrens, and oak-pine barrens (Smith et al. 2002, Plenzler and Michaels 2015, Partridge et al. 2023). In contrast, populations in the eastern parts of its range, where it is in decline, are generally found in disturbed habitats such as powerline rights-of-way and roadsides that are managed for infrastructure (Forrester et al. 2005). Across its geographic range, sundial lupine’s distribution is patchy as many populations are fragmented and isolated from other existing populations (Michaels et al. 2008, Tangren and Frye 2020). Furthermore, this species has a mixed mating system that requires pollination to maximize fitness and has limited seed and pollen dispersal that can limit gene flow between fragmented populations (Ellstrand and Elam 1993, Grigore and Tramer 1996, Shi et al. 2005, Bernhardt et al. 2008).

Due to its conservation status across much of its geographic range, assessment of sundial lupine’s patterns of genetic diversity is important for the management of this species and to inform land managers of ideal propagule sourcing strategies for restoration. Studies investigating population genetics of sundial lupine have been completed in Ohio and Michigan (USA), which have reported moderate-low genetic diversity, significant population structure, and variable inbreeding levels, with one study reporting no significant inbreeding (Shi et al. 2005, Partridge et al. 2023). However, we currently lack an assessment across its entire geographic range. Here, we aim to fill this gap through the genotyping of 316 individuals from 25 populations using nine microsatellite loci. Our specific objectives were to (i) assess the levels of genetic diversity and population structure of sundial lupine throughout its distribution, (ii) estimate levels of inbreeding within populations, and (iii) examine the relationship between inbreeding and genetic diversity with population size and germination rates.

## Methods

### Focal species

Sundial lupine is a perennial legume that blooms from late spring into the summer and typically senesces by July. This species belongs to the North American perennial lineage of the highly diverse genus *Lupinus* (Nevado et al. 2016). The sundial lupine is capable of both sexual reproduction by pollen deposition and asexual reproduction by vegetative propagation. It is able to reproduce sexually through self-pollination, but seed set is greater when pollen is outcrossed between plants (Michaels et al. 2008). Fruits consist of pods with 0–7 seeds that are ballistically dispersed up to 4.8 m or spread by small mammals (Grigore and Tramer 1996, Halpern 2005).

### Sampling

**Figure 1.**
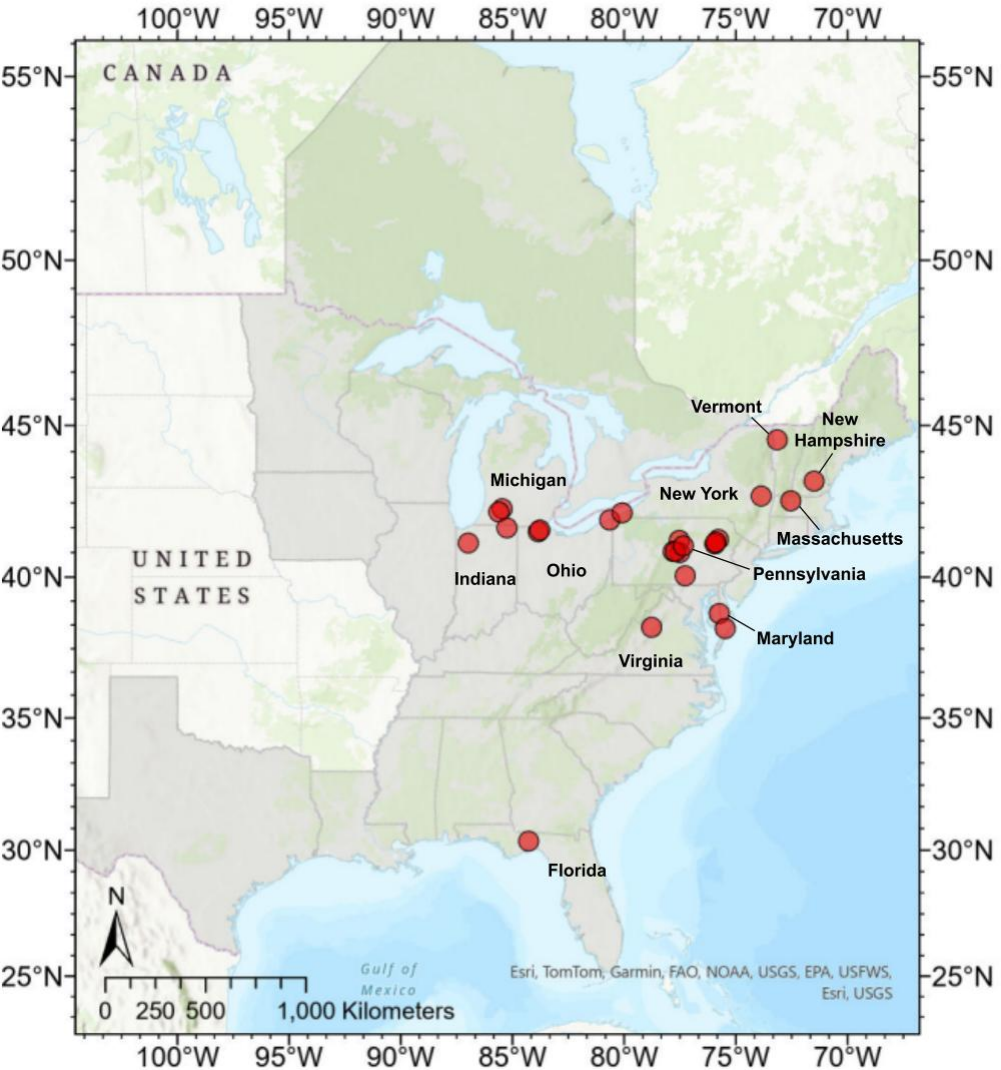
Sampling sites of *Lupinus perennis* for DNA analyses. Leaflets were collected at 25 populations located across 11 states (USA). Collection sites are indicated by circles. Sundial lupine’s distribution is indicated with shading. Map of eastern North America showing the species distribution of sundial lupine and sampling point locations.

We collected leaflet samples from 25 different populations throughout the sundial lupine’s USA range in 2022 (Fig. 1) and obtained all necessary collection permits and permissions prior to collection (Table SI). Our sampling was opportunistic, and we lack equal representation from the northernmost extent and parts of its southern range. Sampling sites ranged in area from 36 m^2^ to a hectare, and sampled habitat types included oak savannas, oak barrens, dunes, and forest edges. We did not gather historical habitat management data for our research sites, but we collected only from sites that are likely natural or unplanted. We personally collected samples from 12 populations in Pennsylvania and from a population in Virginia. Professional ecologists volunteered to collect samples from Indiana, Michigan, New York, Ohio, and Maryland (Fig. 1) based on our protocol and shipped them to us. Samples from Vermont, New Hampshire, Massachusetts, and Florida were collected and shipped to the University of Massachusetts for another research project, and extracted DNA was shared for this project.

In each population, 3–5 leaflets were taken from 15 randomly chosen individual plants at equal distances along a transect. Samples were collected at least 1 m apart to reduce the probability of sampling clones. Less than 15 individuals were collected at two Pennsylvania populations due to restrictions in the amount of available sampling equipment at the time of sampling. After collection from the field, leaf samples were immediately placed in paper coin envelopes and dried in gallon plastic bags containing silica gel to support long-term storage at Penn State University. Populations in Vermont, New Hampshire, Massachusetts, and Florida were sampled by identifying a point in the centre of the population and following a randomly chosen transect to collect two leaflets per individual plant for a minimum of 14 individuals. Leaf samples were dried in silica gel and then stored in a −80°C freezer before DNA extraction.

### DNA extraction and genotyping

We extracted DNA from 289 leaf samples using the Machery-Nagel Nucleo Spin II Plant DNA extraction kit (MACHEREY-NAGEL Inc.) following the manufacturer’s protocol with one modification. Specifically, we eluted DNA on the final step with 55 µl of elution buffer. The average DNA concentration following extraction was 33 ng/µl (SD 24 ng/µl). DNA from 27 individuals that were collected in Vermont, New Hampshire, Massachusetts, and Florida was extracted by Cooper Kimball-Rhines at the University of Massachusetts using Qiagen DNeasy Plant Pro Kit (Cat. No. 69204) and shipped to us for total of 316 individuals in this study. Twenty microsatellite pairs of primers were tested using information reported in Shi (2004), Morris (2009), Gonzalez et al. (2010), Gibbs et al. (2012), Li et al. (2013), and Ricono et al. 2015. After testing, nine loci were used to amplify fragments (Table 1). Forward primers were labelled with four different fluorescent tags, PET, VIC, NED, and 6-FAM to separate loci. We added a six base pair ‘pigtail’ (GTTTCT) to the 5′-end of the reverse primer to reduce stutter in the genotypes (Brownstein et al. 1996). Three multiplex primer sets were designed for more efficient genotyping of all loci and were amplified using the Qiagen Multiplex PCR Kit. PCR was performed in a Mastercycler^®^ pro thermocycler with reaction conditions of 94°C for 5 min; 35 cycles of 94°C for 30 s, 55°C for 30 s, and 72°C for 30 s; followed by a final extension at 72°C for 5 min. PCR products were diluted 1:20 with molecular grade water and sent to the Penn State Genomics Core for fragment size analysis in an Applied Biosystems 3730XL capillary electrophoresis machine with GeneScan LIZ 500 internal standard size. Genotypes were manually scored in Geneious Prime (Version 2023.2).

**Table 1.** Primer sequences used to amplify nine microsatellite loci in *Lupinus perennis*.

Locus	Primer sequence (5′–3′)	Repeat motif	*T*_A_	Allele size (bp)	*N*_A_	*H*_O_	*H*_E_	*M*
GA1^a^	F: TAAGGTTGTGGGGCTTGTCTTC R: CCCATTGAAGAAGAGTTGAAGG	GA(10)	57	133	2	0.03	0.06	1
GAC2^a^	F: ATAGCATCATTGGTGCATGAAG R: ATGTTCTCAACAACCATGCATC	GA(12)	50	193	3	0.04	0.12	1
GAC11^a^	F: TCT TCT ACC AAC GCT CTC AAC C R: TTG AGG CAT TGC AAG AAA GTT G	GA(23)	55	187	6	0.98	0.65	1
GAT5^a^	F: TGTGTGCCATCCATGTCTATTG R: CTTTGAGAGTAATGCGAATCCC	GA(9)	57	193	11	0.37	0.49	1
AATG^a^	F: TGA AAC AAG AAT CGT GAA TGA G R: GGG GAG GTG GGA AAT GAA TAA G	(AGTG)6(AATG)	55	150	5	0.11	0.21	2
GT5^a^	F: CTT GGT CCA GCA AAT GAC TCC R: TCA AAT TCT CAC CGT TGC TGA	(AT)7(GT)10	55	176	3	0.00	0.01	2
GAT7^a^	F: CATTCCTTGTTGTCAGTCCT R: GGAAGGCTTAAGGTACACAG	GA(17)	57	150	4	0.26	0.45	2
LUNA17^b^	F: CGG A TTA GGG TTT GGG TTT T R: AAA GAT GGA TGA GGC AAA GG	(TTG)4(GTT)6	55	232–238	4	0.29	0.4	3
LUP4^c^	F: AGC AGA GAC TCG AAT AGG TGA GA R: TTG GAG CAG AAA GAT CAG GAA	CT(10)	55	130	7	0.36	0.6	3

The table includes locus name, primer sequences (5′–3′), repeat motif, annealing temperature (*T*_A_) in Celsius degrees (°C), allele size range (bp), number of alleles (*N*_A_), observed heterozygosity (*H*_O_), expected heterozygosity (*H*_E_), and assigned multiplex group (1–3) (*M*).

^a^Primers designed by Shi (2004).

^b^Primers designed by Morris (2009).

^c^Primers designed by Li et al. (2013).

### Population genetic analysis

We performed all estimations of genetic diversity in R Statistical Software (v4.2.2; R Core Team 2022). We assessed null allele frequencies using Chakraborty et al. (1994)’s method with the package *PopGenReport* (Adamack and Gruber 2014) and probability of identity (PID) was assessed with the package *PopGenUtils* using function ‘pid_calc’ (Tourvas et al. 2023). To test whether populations and loci were in Hardy–Weinberg equilibrium, we used the function ‘hw.test’ in the package *pegas* with χ^2^ comparisons (Paradis 2010). We tested for linkage disequilibrium using the *poppr* package (Kamvar et al. 2014). Genetic diversity indices and summary statistics were calculated using *poppr* and *hierfstat* packages and included observed and expected heterozygosity (*H*_O_, *H*_E_), number of alleles per locus (*N*_A_), allelic richness (*A*_R_), the inbreeding coefficient (*F*_IS_), and the percentage of missing data. For estimates of *F*_IS_, we obtained estimates of 97.5% confidence intervals with a bootstrap approach of 500 replicates. We calculated rarefied allelic richness to account for differences in sample size for populations that had a minimum of 10 individuals sampled after we removed clones. We obtained estimates of rarified allelic richness using *hierfstat* package (Goudet 2004).

We quantified summary statistics of population genetic structure with two pairwise G-statistics: Nei’s *G*_ST_ and Hedrick’s *G′*_ST_ using the function ‘diff_stats’ in the package *mmod* (Winter 2012; Table SIII). Hedrick’s *G*′_ST_ is suited for polymorphic genetic markers and is therefore reported as our measure of genetic distance (Hedrick 2005). Isolation by distance was evaluated using a Mantel test to examine the relationship between genetic distance (Hedrick's *G′*_ST_) and geographic distance using the R package *adegenet.* Genetic clusters were inferred using Bayesian clustering implemented in STRUCTURE version 2.3.4 (Pritchard et al. 2000). We conducted 10 independent runs of each value of *K* for *K* = 1–18. Markov Chain Monte Carlo simulation values were set for a burn-in period of 10 000 iterations and a run length of 100 000 iterations in an admixture model with correlated allele frequencies among populations. StructureSelector was used to estimate the optimal *K* based on Evanno’s Δ*K* approach (Evanno et al. 2005) and Puechmaille’s four-estimator approach (MedMedK, MedMeaK, MaxMed K, MaxMeaK) (Puechmaille 2016). Due to our uneven sampling, we report the results from Puechmaille’s approach. Structure Plot V2.0 was used to visualize membership probability to each cluster (Ramasamy et al. 2014). We visualized the admixture map using the online tool mapmixture 1.2.0 (Jenkins 2024). We visualized population differentiation through a discriminant analysis of principal components (DAPCs) using the package *adegenet* to identify genetic clusters (Jombart 2008). An analysis of molecular variance (AMOVA) was performed to test for significant population genetic differentiation between assigned genetic clusters determined by STRUCTURE analysis and populations of sundial lupine’s range using the function ‘poppr.amova’ in the package *poppr* (Kamvar et al. 2014). Significance was tested with 1000 permutations and two strata were used for the AMOVA test: cluster and population.

### Measures of population size and germination

We obtained estimates of population size for each collection site. In populations outside of Pennsylvania, volunteers estimated population size through visual counts. Due to the large size of some populations, some of these estimates were proxies based on area rather than individual counts. In Pennsylvania (USA), we measured population sizes and germination success for six populations (PA1S, PA1C, PA2C, PA4C, PA3NE, and PA4NE). We counted all aboveground lupine clumps, defined as clustered groups of inflorescences, to assess population size. To quantify germination success, seeds were collected from 20 randomly chosen plants per population in summer 2022 and sent to Mt. Cuba Center, a botanic garden in Delaware (USA), for germination. Seeds were scarified by soaking in water initially heated to 88°C for 24 h and then mixed with rhizobia. Following scarification, seeds were sown in ProMix FPX (PRO-MIX^®^) and placed on a bench heated to 21°C. Soil was kept consistently moist, and per cent germination was recorded after 1 month. Individual germination rates were averaged by population before analysis.

We assessed relationships between population size and genetic diversity metrics, allelic richness and expected heterozygosity with linear models. To normalize population size, we used log population size for all analyses. For populations in which percent germination was collected, we used linear models to test the association between inbreeding coefficients, allelic richness or expected heterozygosity, and germination success.

### Ethics approval

The species sampled is not endangered or protected. Fieldwork was conducted in locations where permission was granted and necessary collection permits were obtained.

## Results

### Genotyping

All of the tested microsatellite loci were polymorphic with the exception of locus GT5. Although sampling was designed to limit the collection of clones, we identified 50 individuals that shared a multilocus genotype (MLG) with at least one other sampled individual out of the 316 total individuals included (Table SII). We found that there is a 0.1% chance that two unrelated individuals shared an MLG based on our allele frequencies and that loci GT5 (0.97) and GA1 (0.89) had high probability of identity, while GAC11 (0.20) and LUP4 (0.24) had low values, indicating more discrimination between individuals. Clones were removed, and a total of 266 individuals were used in analysis (Table 2). Among the 266 individuals examined, 10% of genotypes were missing within the data and missing data per locus ranging from 3% (locus LUNA17) to 22.2% (locus AATG). Alleles per locus ranged from two in locus GA1 to 11 in locus GAT5. Null alleles were present in all loci ranging between −0.21 (GAC11) and 0.51 (GAC2). We observed significant linkage disequilibrium between loci (p.rD = 0.0240), with the strongest correlation observed between loci AATG and GAT5 (rbarD = 0.121). None of the loci were at Hardy–Weinberg Equilibrium across all populations. At the population level, 12 out of 25 exhibited all loci at equilibrium, 10 showed 1 locus violating assumptions of equilibrium, and 3 showed 2 or more loci in disequilibrium. Locus GAC11 was in disequilibrium for 11 populations, but analyses with and without it yielded consistent results, so it was included in all analyses.

**Table 2.** Population genetic diversity statistics for *L. perennis* based on nine microsatellite markers.

Population	State	*N*	*n*	*A*	*A*_R_	*A*_P_	*H*_O_	*H*_E_	*F*_IS_	97.5% CI
FL1	FL	8	7	23	2.56	20	0.44	0.44	−0.03	(−0.286 to 0.211)
VA1	VA	15	13	16	1.78	0	0.18	0.21	0.18	(−0.572 to 0.734)
MD1	MD	15	14	19	2.11	1	0.28	0.31	0.23	(−0.192 to 0.555)
MD2	MD	15	12	26	2.89	1	0.33	0.36	0.13	(−0.187 to 0.312)
NY1	NY	15	13	19	2.11	0	0.26	0.28	0.17	(−0.381 to 0.566)
MA1	MA	8	7	15	1.67	0	0.29	0.24	−0.20	(−0.750 to 0.114)
NH1	NH	5	5	14	1.56	0	0.26	0.23	−0.15	(−0.750 to 0.395)
VT1	VT	6	5	15	1.67	0	0.31	0.26	−0.20	(−0.699 to 0.2)
PA1S	PA	15	13	14	1.56	0	0.18	0.17	0.14	(−0.471 to 0.699)
PA1C	PA	15	12	17	1.89	0	0.21	0.22	0.04	(−0.665 to 0.581)
PA2C	PA	15	12	15	1.67	0	0.21	0.21	0.01	(−0.612 to 0.524)
PA3C	PA	15	12	19	2.11	0	0.3	0.26	−0.01	(−0.399 to 0.278)
PA4C	PA	15	14	16	1.78	0	0.3	0.28	−0.10	(−0.440 to 0.167)
PA5C	PA	15	10	13	1.44	0	0.16	0.17	0.08	(−0.818 to 0.543)
PA1NE	PA	15	15	20	2.22	1	0.41	0.33	−0.18	(−0.496 to 0.013)
PA2NE	PA	7	5	14	1.56	0	0.27	0.29	−0.12	(−0.822 to 0.755)
PA3NE	PA	8	8	17	1.89	0	0.41	0.33	−0.26	(−0.577 to 0.036)
PA4NE	PA	15	14	20	2.22	2	0.3	0.3	0.15	(−0.341 to 0.443)
PA1W	PA	15	13	16	1.78	0	0.27	0.24	−0.10	(−0.436 to 0.365)
OH1	OH	14	13	15	1.67	0	0.33	0.24	−0.27	(−0.690 to 0.197)
OH2	OH	15	14	16	1.78	1	0.25	0.22	−0.08	(−0.389 to 0.032)
MI1	MI	10	5	14	1.56	0	0.19	0.18	−0.13	(−0.499 to 0)
MI2	MI	10	7	15	1.67	0	0.25	0.24	0.03	(−0.602 to 0.653)
IN1	IN	15	12	17	1.89	2	0.22	0.25	0.21	(−0.469 to 0.621)
IN2	IN	15	11	17	1.89	0	0.22	0.22	0.14	(−0.374 to 0.479)

Table includes population abbreviation, state collected, geographic region within species range, number of individuals collected per population (*N*), number of individuals used in analysis after removing clones (*n*), total number of alleles (*A*), allelic richness (*A*_R_), private alleles (*A*_P_), observed heterozygosity (*H*_O_), expected heterozygosity (*H*_E_), inbreeding coefficients (*F*_IS_), and 97.5% confidence intervals for inbreeding coefficients (*F*_IS_) (97.5% CI).

### Genetic diversity estimates

Population averages of allelic richness ranged from 1.44 to 2.56, while estimates of rarefied allelic richness within populations that had >10 individuals used in our analysis, ranged from 1.72 to 3.22. Expected heterozygosity estimates ranged from 0.17 to 0.44 and averaged 0.26 (Table 2). The lowest estimate was obtained in population PA1S and the highest in FL1. Seven populations contained private alleles, with the southernmost population from Florida (FL1) containing 17 and IN1, MD1, MD2, PA1NE, PA4NE, and OH2 containing 1 or 2 private alleles (Table 2).

### Population genetic structure

Overall, we found that populations of sundial lupine show genetic differentiation with a global Hedrick’s *G*′ = 0.31. For pairwise estimates, Hedrick’s *G*′_ST_ was greatest between population FL1 in Florida and IN2 in Indiana (0.63) and lowest between population MD1 in Maryland and NH1 in New Hampshire (0.003; Table SIII). We found a significant pattern of isolation-by-distance to describe genetic differentiation between populations (*R*^2^ = 0.30, *P* = 0.036, Fig. S1).

**Figure 2.**
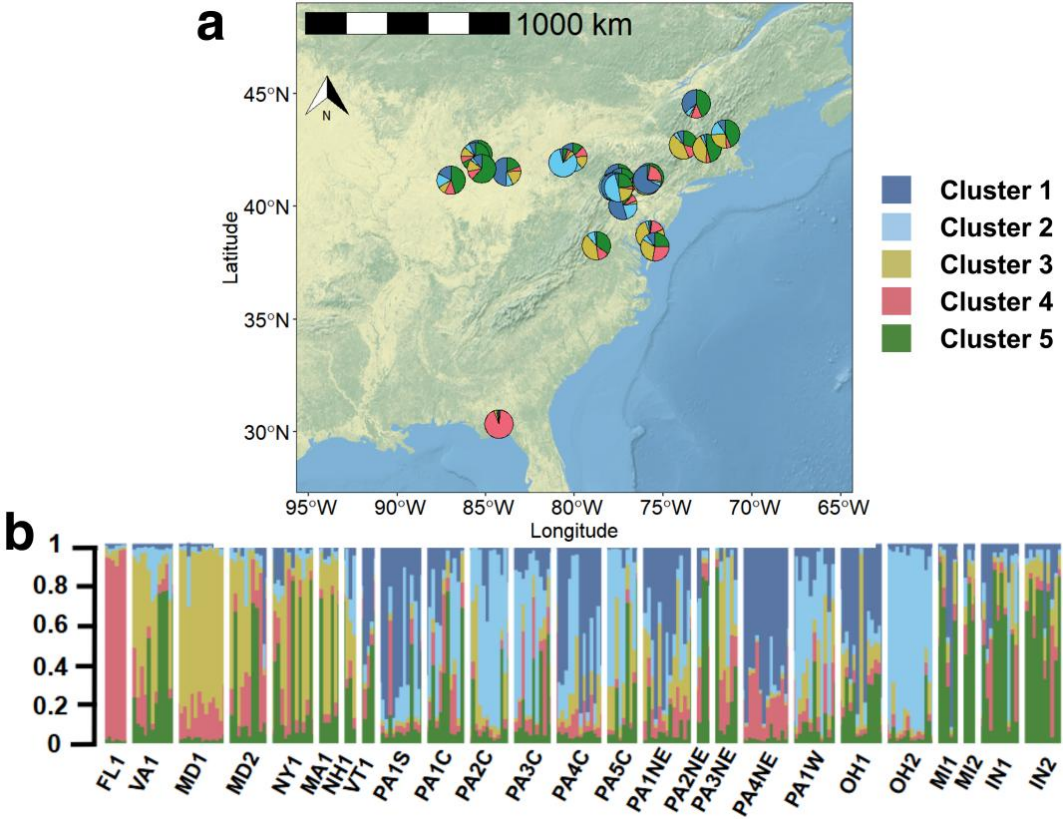
Population structure of 266 samples of sundial lupine described at *K* = 5. a) The map depicts the probability of genetic cluster assignment across the 25 sampled populations. Probability is described by up to five colours within-population pie charts. b) The bar plot shows individuals grouped by populations based on microsatellite data. Each vertical bar represents an individual and the corresponding unique colour represents the assigned genetic cluster. Membership probability to a genetic cluster is shown by the length of the bar for each individual. Graphical representation of sundial lupine population structure showing membership probability to five genetic clusters for each sampled individual and population.

For the Bayesian clustering analysis, the Evanno’s Δ*K* method identified two genetic clusters and Puechmaille’s four-estimator approach identified 5 (MedMed K, MedMean K, MaxMean K) or 7 (MaxMed K) genetic clusters, a more accurate estimate given our uneven sampling size (Puechmaille 2016; Fig. S5). We report five clusters based on the results from the four-estimator approach. The STRUCTURE results, using assignment to five clusters, showed high admixture but structure is partially explained by geography (Fig. 2). The southernmost population, FL1, shows high assignment to Cluster 4 (0.9). While other populations contain more admixture, there is generally similar cluster assignment within regions. Three southern populations (VA1, MD1, MD2) show the highest assignment to Clusters 3 and 4, and populations in central (PA2C, PA3C) and western (PA1W) Pennsylvania cluster with an eastern population in Ohio (OH2) in Cluster 2. Midwestern populations show assignment to Cluster 5 (MI1, MI2, IN1, IN2), in addition to three northeastern populations (MA1, VT1, NH1) that also show high assignment to Cluster 3. Clustering assigned with DAPC varied from the STRUCTURE analysis (Fig. S2). The greatest decrease of the Bayesian information criterion value followed *K* = 7 (Fig. S3), and clearly showed a distinction of the southernmost population belonging to a different cluster than midwestern and northeastern populations. The results of AMOVA indicate that genetic differentiation is significant between clusters assigned based on STRUCTURE results at *K* = 5 (*P* = 0.01), populations (*P* = 0.01), and populations within a cluster (*P* = 0.01). Clusters accounted for 20.67% of the variation, while populations within clusters 17.12%, and within populations 62.19% of variation (Table 3).

**Table 3.** Results of AMOVA and genetic variation within and among clusters and populations of sundial lupine.

Variation	Sum of squares	Sigma	% variance explained	*P*-value
Among clusters	134.73	0.561	20.69	0.01
Among populations	213.51	0.464	17.12	0.01
Within populations	335.76	1.687	62.19	0.01

### Inbreeding estimates, population size, and germination

We found that no populations showed evidence of inbreeding. Inbreeding coefficients ranged from −0.27 (−0.690 to 0.197) to 0.23 (−0.192 to 0.555), with a Maryland population (MD1) being the most positive. Population size estimates ranged from 20 to 10 000 individuals with a median of 400 individuals. No measures of genetic diversity were correlated with population size (*F*_1,22_ = 0.94, *P* = 0.343; *F*_1,22_ = 0.0013, *P* = 0.972 for allelic richness and expected heterozygosity, respectively). Similarly, we observed no relationship between inbreeding coefficients and log population size (*F*_1,22_ = 0.946, *P* = 0.341).

Germination success from six Pennsylvania populations ranged from 79.2% to 89.6% (mean 84.47, SD 4.62). We observed a positive relationship between germination success and inbreeding coefficients (*F*_1,4_ = 8.41, *R*^2^ = 0.68, *P* = 0.044; Fig. S4). However, we found no relationship between germination success and allelic richness or expected heterozygosity (*P* = 0.451, *P* = 0.478).

## Discussion

The results of this study reveal low genetic diversity in populations of sundial lupine but no evidence of inbreeding. Furthermore, we detected significant genetic differentiation among populations with five genetic groups across the species’ range but significant admixture between these clusters. Overall, these findings indicate that propagules can be sourced from different populations and planted to better conserve this species in areas where their populations are declining. Below, we describe these findings in greater detail and compare them with previous studies focused on plants of conservation concern.

### Genetic diversity

Our results show that populations of sundial lupine exhibit low genetic diversity across the species’ geographic range. Diversity metrics were relatively similar across all populations that were sampled and were generally low. We observed an average allelic richness of 1.77 without rarefaction. Plants of conservation concern, including those in fragmented habitats, can present this pattern indicating that the species is likely more vulnerable to environmental stressors and has less adaptive potential (Aguilar et al. 2008). For example, an investigation of a threatened annual species, *Leavenworthia exigua* Rollins var. *laciniata*, reported low observed heterozygosity (Edwards et al. 2022). Similarly, *Pulsatilla patens* (L.) Mill., a rare perennial species, showed low allelic richness estimates across 29 populations (Szczecińska et al. 2016). In contrast, a study on kincaid’s lupine (*Lupinus oreganus* A. Heller var. *kincaidii*), a threatened perennial species, reported high levels of genetic diversity likely due to its long lifespan, despite its habitat being fragmented (Severns et al. 2011).

Allele counts per population were similar in the northeast and west of the Appalachians but highest in the southernmost population. Previous investigation of eight sundial lupine populations in Michigan reported an average allelic richness of 2.34 alleles per population, which is greater than our average estimate (Partridge et al. 2023). Comparisons of average alleles per locus exist from three other studies that investigated sundial lupine genetics. Shi (2004), Gibbs et al. (2012), and Partridge et al. (2023) all amplified two of the same microsatellite loci (GAC2 and GAT5) and populations within several of the same states (Ohio, New York, and Michigan) as our study. Our estimates of average alleles per locus are lower than those reported in previous studies, likely due to our smaller sample sizes.

### Genetic structure

We found the presence of five genetic clusters with populations showing significant admixture across sundial lupine’s range partially explained by the Appalachian Mountains acting as a geographic barrier. East of the Appalachian Mountains, populations (FL1, MD1, MD2, VA1, and NY1) have higher assignments to Cluster 4 or 3. Within the mountain range, in central Pennsylvania, most populations tend to have higher assignments to Cluster 1, while some Pennsylvania and Ohio populations were primarily assigned to Cluster 2. Other sampled populations west of the Appalachian Mountain range tend to be assigned to Cluster 5 (MI1, MI2, IN1, IN2), which is also true for northeastern populations (MA1, VT1, NH1). Partridge et al. (2023) identified two genetic clusters in Michigan populations containing admixture. Geographic barriers such as the Appalachian Mountains that span through the eastern part of sundial lupine’s range may be acting as a barrier to gene flow between regional genetic clusters (Soltis et al. 2006, Barnard-Kubow et al. 2015). Sundial lupine lacks long-distance seed dispersal, although seeds could be moved short distances by rodents or small mammals. Because sundial lupine habitat is often fragmented, and populations are separated at distances that spatially prevent both pollen and seed dispersal, it is likely that the current patterns of admixture are due to large effective population sizes and more connected populations of this species in the past.

Our broad patterns of genetic diversity pose some hypotheses about potential biogeographic scenarios that have shaped the spatial distribution of the genetic diversity of this species. The Appalachian Mountain range is an important phylogeographic barrier for plants and animals in eastern North America (Soltis et al. 2006). We hypothesize that sundial lupine’s geographic range was constrained to southern areas during glaciations, and it recolonized its current range from the south from two different refugia east and west of the Appalachians. This interpretation is supported by the high differentiation of the population from Florida (FL1) compared with the rest of the distribution. However, our sampling may bias this differentiation since we lacked much sampling in the southern extent of its range. The difference between western and eastern populations is also apparent by the differentiation of assignment to Cluster 3, with higher membership probabilities observed in the east. In western North America, *Lupinus lepidu*s Douglas ex Lindl. is suspected of colonization from southern populations after glaciations (Weitemier 2000). Furthermore, diversification rates estimated with chloroplast DNA suggest sundial lupine is most closely related to Andean-Mexican *Lupinus* species that are south of its current range (Drummond 2008). It is important to note that some of the observed patterns of population structure may have been influenced by human activity. Western populations and northeastern populations exhibit a high proportion of ancestry from Cluster 5, which may be explained by human-mediated plantings from eastern populations. Partridge et al. (2023) describe habitat management that involved seeding in central-west Michigan. While authors describe that seeding was done from local propagules, it is possible that within the populations we studied, propagules could have been sourced from northeastern populations or vice versa. While we do not have information regarding seeding history at our sites, it is known that seeding is a management tool that is used to enhance sundial lupine populations, especially in the Midwest where there are stronger recovery efforts for the Karner Blue Butterfly (Kleintjes et al. 2003; Reinhardt et al. 2017). Additionally, another anthropogenic-mediating factor obscuring population structure is possible hybridization with the western species *Lupinus polyphyllus* Lindl., which was introduced to eastern North America in the mid-1900s (Gleason and Cronquist 1991, Gibbs et al. 2012). Thus, it is possible that human-mediated seed dispersal may have mixed individuals between populations.

### Inbreeding and germination

Populations showed inbreeding coefficients not significantly different from zero, similar to a study investigating sundial lupine in Michigan (Partridge et al. 2023). Because sundial lupine benefits from outcrossing by pollinators, populations lacking robust pollinator communities may rely on self-pollination or invest in clonal rhizome production (Francis 2017). Floral density drives pollinator visitation to sundial lupine so we expected that populations with lower floral abundance would have increased inbreeding coefficients due to a lack of pollinator-mediated outcrossing (Bernhardt et al. 2008). However, we found no evidence of significant inbreeding coefficients in any of the studied populations indicating that despite the small size and low levels of genetic diversity in some populations, they may be avoiding inbreeding due to their mixed mating strategy, long lifespan, or sufficient pollinator-mediated outcrossing (Goodwillie et al. 2005, Severns et al. 2011, Devaux et al. 2014). Although we did not observe levels of inbreeding that significantly deviated from zero, we found that populations with an excess of heterozygous showed lower germination success (Fig. S4). While our sample size is limited to draw definite conclusions, this pattern indicates the potential for reduced fitness if individuals from genetically distinct populations are crossed. Ultimately, both inbreeding and outbreeding depression should be evaluated when considering genetic rescue for rare plant species.

### Implications for species conservation

Our genetic analysis suggests that southern populations and midwestern populations have the most private alleles and should be considered areas of high conservation value. When conserving sundial lupine, or other rare species, management of existing populations should take priority over attempting to establish new populations (Michaels et al. 2019). For sundial lupine, reaching and maintaining at least 500 individuals (e.g. via supplemental planting, applying prescribed fire, and removing encroaching trees) is recommended (Michaels et al. 2019, Petitta et al. 2023). However, when supplemental planting is necessary, the genetic clustering described here should be considered to identify populations to source seed. Population genetic variation and adaptive potential could be improved by sourcing seeds from alternate genetic clusters. However, land managers should consider local adaptation to regional habitats and use caution against long-distance movement between populations in the extreme ranges of the species. Additionally, seed sources should be scrutinized. Gibbs et al. (2012) examined commercially available ‘sundial lupine’ seeds and discovered that some marketed seeds were interspecific hybrids that had decreased survival in a common garden experiment. These hybrids are suspected to be Russell hybrids, a cross with *L. polyphyllus*, which has escaped cultivation in the northeastern United States (Gleason and Cronquist 1991, Gibbs et al. 2012). Managers should also consider the local climate and potential regional environmental adaptation when considering moving propagules between distant populations.

Ultimately, we recommend further analysis of sundial lupine population structure, especially for populations at the northern extent of its range in southern Canada. Further information regarding the population genetics of sundial lupine is an important component to its conservation and serves as a model for other oak savanna–associated plant species of conservation concern. The results reported in this study provide the first investigation of sundial lupine population structure throughout most of its range and serve as a guide to inform the survival of this species.

## Supplementary Material

plaf047_Supplementary_Data

## Data Availability

All the raw datasets mentioned in the manuscript are publicly available on Github (https://github.com/isapeta/PopGen_Data).
