# Supplementary material for: Assessing the status of sundial lupine (Lupinus perennis L.) genetic diversity and population structure throughout its distribution
Source: AoB Plants. 2025 Sep 2;17(5):plaf047. doi: 10.1093/aobpla/plaf047 (PMC12449230; doi:10.1093/aobpla/plaf047)
Supplement: plaf047_Supplementary_Data [file plaf047_supplementary_data.zip › Supporting_PetittaEtAl.docx.pdf]

Supporting Figures

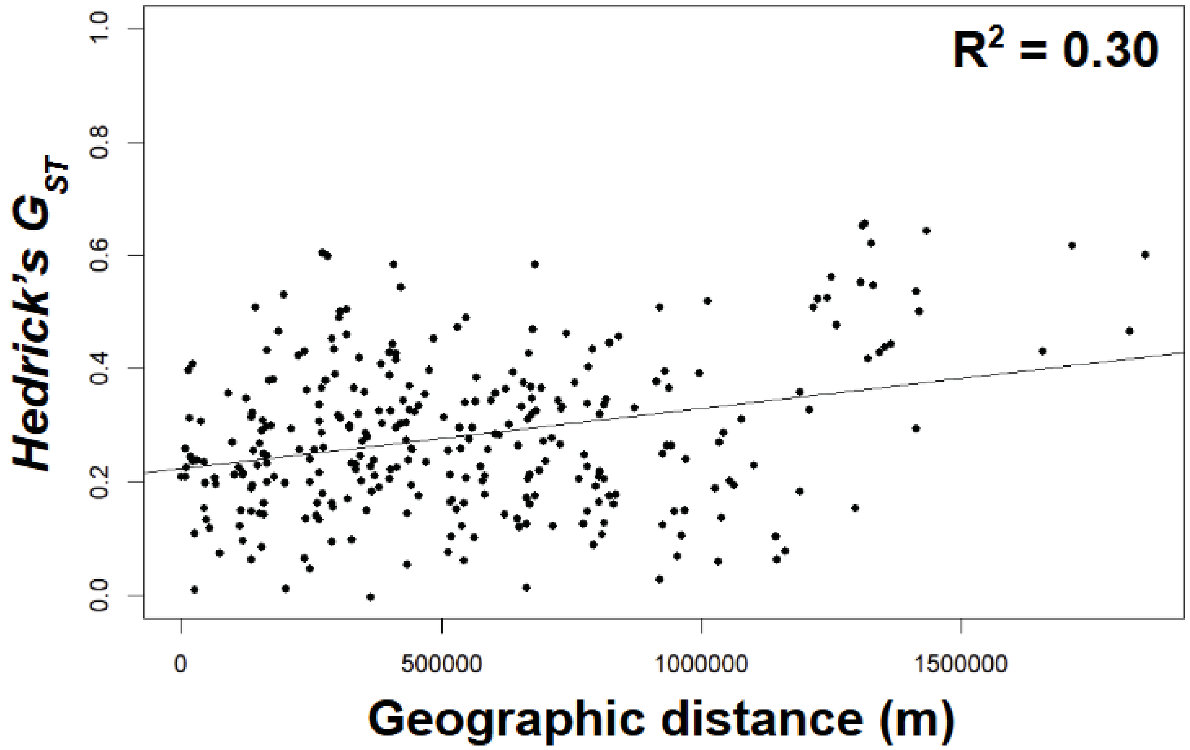

Figure S1. Correlation of genetic distance (pairwise Hedrick's G'_{ST}) and geographic distance (meters). Black points represent pairwise differences of sundial lupine populations. Significance was tested with a Mantel test. P = 0.036.

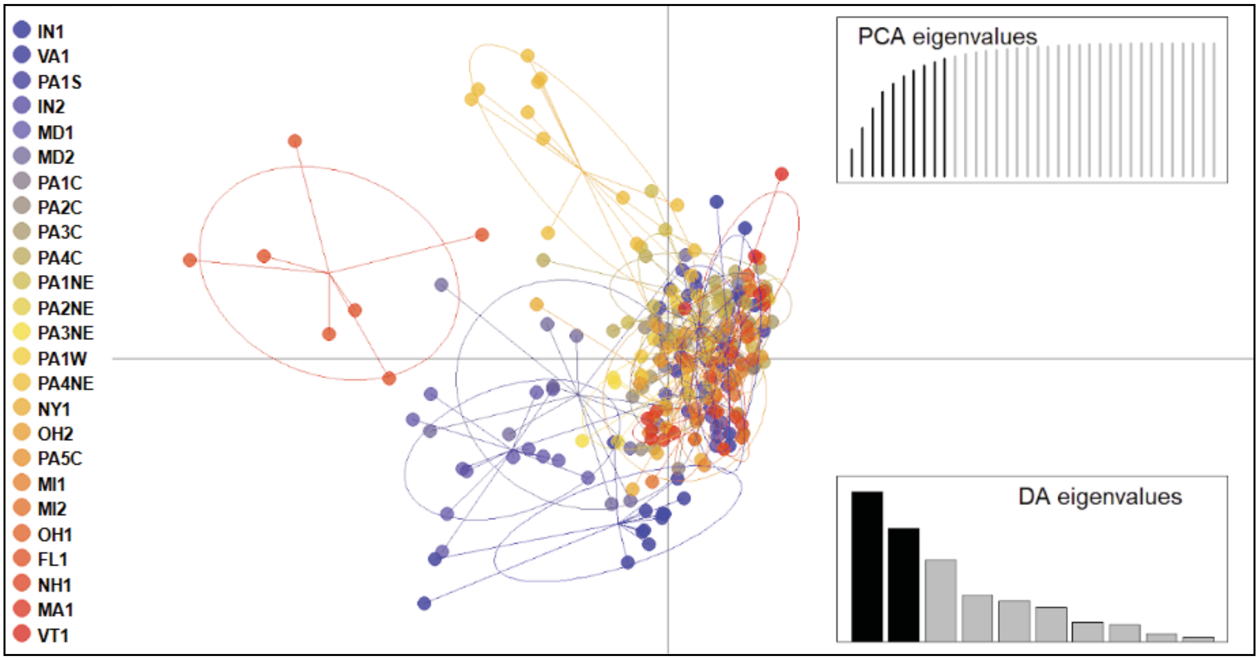

Figure S2. Clustering scatter plot based on Discriminate Analysis of Principal Components (DAPC) at K = 7. Points indicate sampled individuals and circles indicate populations. Populations are differentiated by color.

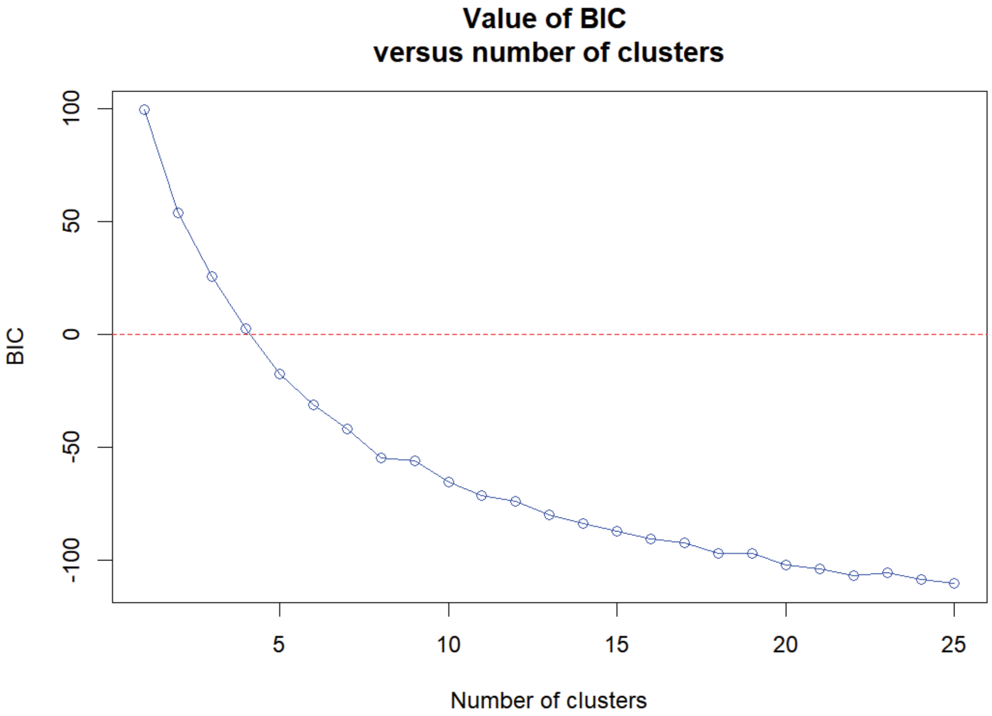

Figure S3. Bayesian information criterion (BIC) plot at K = 7.

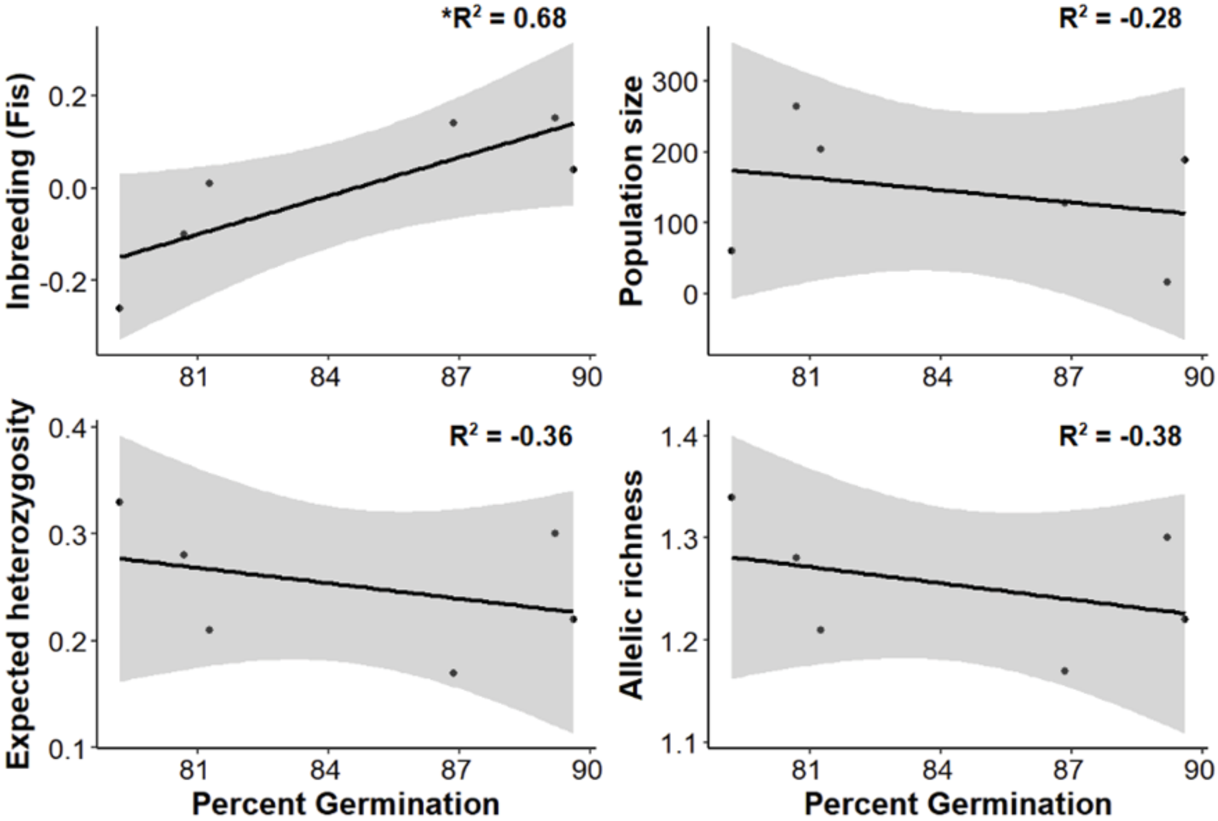

Figure S4. Correlation of inbreeding coefficients, population size, expected heterozygosity, allelic richness, and percent germination of six sundial lupine populations in Pennsylvania. Black points represent population averages. Shading represents 95% confidence intervals. Significance was tested with a linear model. Significant relationships are indicated with an asterisk (P = 0.044).

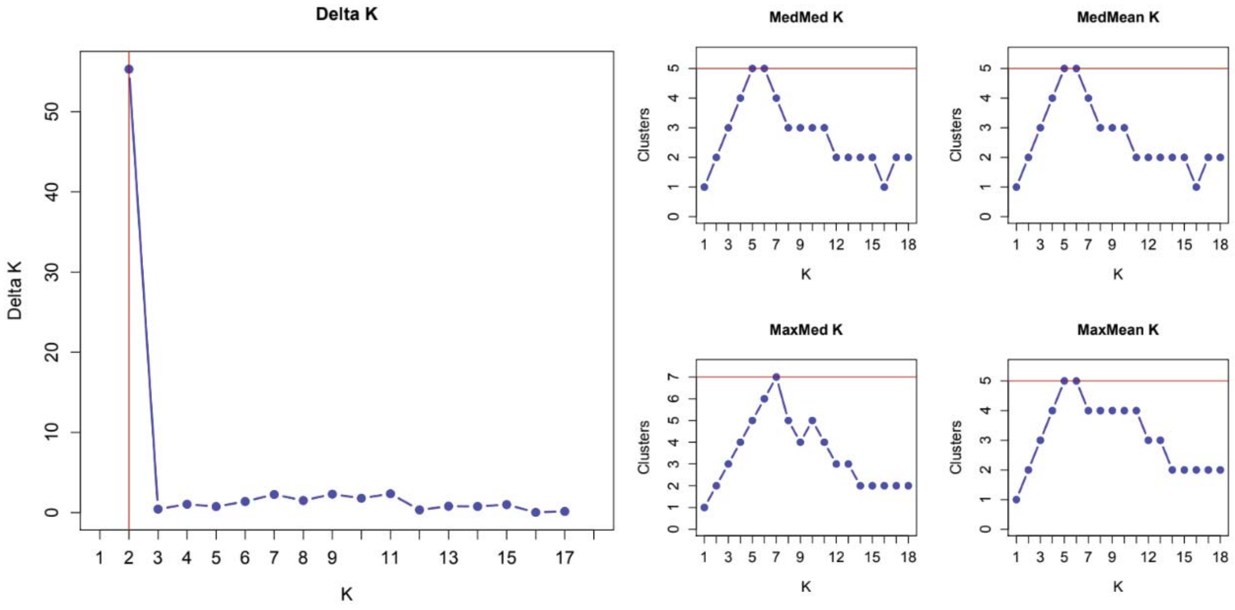

Figure S5. Delta K, MedMed K, MedMean K, MaxMed K, and MaxMean K based on STRUCTURE output for K=1-18.

Supporting Tables

Table SI. Permits required for sample collection. Samples collected in Maryland, Ohio, Michigan, and New York did not require a permit. Samples collected in Florida, Massachusetts, New Hampshire, and Vermont were organized by Cooper Kimball-Rhines and extracted DNA was shared for our study, therefore we did not lead the permitting process for those states. Required permits were acquired by Cooper Kimball-Rhines for these populations.

State	Permitting agency	Permit number	Collector
Indiana	Department of Natural Resources	NP22-31	Scott Namestnik
Pennsylvania	Department of Conservation and Natural Resources: Bureau of Forestry	SFRA-2232	Isabella Petitta
Pennsylvania	Game Commission	55835 and 55346	Isabella Petitta
Pennsylvania	Department of Conservation and Natural Resources: Bureau of State Parks	2022-46	Autumn Sabo
Virginia	US National Park Service	SHEN-2022-SCI-0008	Isabella Petitta

Table SII. List of repeating multilocus genotypes (MLG) or clones. Individuals are coded with their origin populations and followed by their individual number code. Clones that were repeated were removed, leaving only one copy of each MLG. Bold individuals were removed from the dataset and not included in the analysis.

Individual	Clone 1	Clone 2	Clone 3	Clone 4	Clone 5
**IN1-21**	IN1-23				
**IN1-25**	OH1-293				
**IN1-27**	IN2-64	**IN2-69**	**IN2-71**	**PA1C-109**	**MI1-271**
**VA1-37**	VA1-40				

**VA1-42**	VA1-44				
**PA1S-46**	PA4C-163				
**PA1S-52**	PA1S-56				
**IN2-63**	OH1-302				
**IN2-72**	MA1-336	**MA1-337**			
**MD1-81**	IN2-62	**MD2-91**	**NY1-236**		
**MD2-102**	MI1-276				
**MD2-104**	IN2-75	**PA1C-119**	**MI1-274**	**MI1-275**	**MI2-287**
**PA1C-114**	PA2C-133	**PA3C-147**	**OH2-254**		
**PA2C-130**	PA5C-256				
**PA2C-131**	PA2C-128	**PA5C-270**			
**PA2C-132**	PA2C-134				
**PA3C-139**	PA2C-126	**PA3C-145**	**PA1W-210**		
**PA4C-160**	PA4C-162				
**PA2NE-181**	PA2NE-182				
**PA2NE-183**	PA2NE-187				
**PA1W-209**	OH2-248				
**PA4NE-221**	PA4NE-222				
**NY1-227**	OH1-297				

**PA5C-256**	PA5C-257		
**PA5C-258**	PA5C-267		
**PA5C-264**	**MI1-278**	**MI2-289**	NH1-331
**PA5C-265**	PA5C-269		
**MI1-277**	OH1-301	**OH1-303**	
**MI2-288**	NH1-335		
**FL1-324**	FL1-325		
**VT1-345**	VT1-346		

Table SIII. Pairwise genetic distance between populations. Pairwise Nei's G_{ST} (upper diagonal) and Hedricks's G'_{ST} (lower diagonal) values for the 25 populations of sundial lupine.

**GST**	**IN1**	**VA1**	**PA1S**	**IN2**	**MD1**	**MD2**	**PA1C**	**PA2C**	**PA3C**	**PA4C**	**PA1NE**	**PA2NE**	**PA3NE**	**PA1W**	**PA4NE**	**NY1**	**OH2**	**PA5C**	**MI1**	**MI2**	**OH1**	**FL1**	**NH1**	**MA1**	**VT1**
**IN1**	0.000	0.128	0.106	0.032	0.245	0.072	0.023	0.106	0.043	0.122	0.099	0.114	0.073	0.100	0.147	0.094	0.123	0.110	0.005	0.073	0.059	0.217	0.035	0.120	0.009
**VA1**	0.295	0.000	0.269	0.108	0.186	0.060	0.156	0.175	0.118	0.233	0.198	0.232	0.072	0.165	0.294	0.132	0.168	0.135	0.059	0.161	0.196	0.229	0.070	0.113	0.206
**PA1S**	0.241	0.523	0.000	0.182	0.338	0.159	0.090	0.150	0.097	0.091	0.085	0.205	0.198	0.106	0.119	0.232	0.139	0.235	0.197	0.200	0.060	0.278	0.204	0.230	0.096
**IN2**	0.080	0.249	0.382	0.000	0.199	0.028	0.068	0.170	0.061	0.152	0.094	0.134	0.048	0.144	0.183	0.052	0.181	0.143	-0.032	0.010	0.060	0.221	0.003	0.055	0.090
**MD1**	0.546	0.424	0.665	0.452	0.000	0.100	0.263	0.221	0.235	0.198	0.165	0.269	0.136	0.230	0.289	0.126	0.250	0.200	0.226	0.191	0.203	0.183	0.146	0.138	0.276
**MD2**	0.193	0.157	0.372	0.077	0.271	0.000	0.090	0.134	0.069	0.128	0.093	0.120	0.047	0.091	0.140	0.035	0.138	0.125	0.023	0.027	0.079	0.109	0.026	0.029	0.104
**PA1C**	0.059	0.343	0.205	0.164	0.565	0.232	0.000	0.084	0.014	0.105	0.107	0.080	0.070	0.076	0.148	0.116	0.091	0.116	0.048	0.106	0.082	0.191	0.059	0.170	0.035
**PA2C**	0.250	0.378	0.322	0.371	0.491	0.331	0.198	0.000	0.087	0.071	0.112	0.158	0.125	0.057	0.218	0.148	0.031	0.015	0.159	0.209	0.121	0.237	0.083	0.185	0.128
**PA3C**	0.110	0.276	0.224	0.150	0.531	0.186	0.037	0.210	0.000	0.115	0.092	0.084	0.075	0.064	0.154	0.112	0.045	0.103	0.030	0.106	0.082	0.187	0.056	0.103	0.073
**PA4C**	0.295	0.501	0.215	0.352	0.470	0.332	0.253	0.177	0.282	0.000	0.019	0.127	0.144	0.072	0.122	0.109	0.105	0.110	0.187	0.135	0.036	0.233	0.105	0.164	0.099
**PA1NE**	0.253	0.454	0.210	0.236	0.417	0.259	0.267	0.277	0.239	0.308	0.000	0.131	0.104	0.107	0.097	0.101	0.129	0.136	0.127	0.089	0.004	0.227	0.091	0.112	0.097
**PA2NE**	0.275	0.491	0.431	0.310	0.593	0.308	0.194	0.355	0.208	0.365	0.329	0.000	0.090	0.092	0.158	0.088	0.147	0.185	0.160	0.087	0.146	0.190	0.100	0.203	0.075
**PA3NE**	0.191	0.186	0.443	0.127	0.355	0.137	0.180	0.306	0.199	0.182	0.285	0.236	0.000	0.108	0.169	0.055	0.154	0.117	0.040	0.069	0.121	0.127	0.030	0.128	0.085
**PA1W**	0.242	0.367	0.242	0.327	0.517	0.238	0.184	0.141	0.162	0.307	0.272	0.226	0.275	0.000	0.133	0.098	0.045	0.093	0.134	0.136	0.107	0.187	0.086	0.147	0.078
**PA4NE**	0.354	0.611	0.278	0.418	0.645	0.365	0.349	0.481	0.371	0.275	0.259	0.377	0.424	0.322	0.000	0.179	0.217	0.274	0.208	0.150	0.110	0.210	0.202	0.227	0.086
**NY1**	0.235	0.311	0.486	0.131	0.317	0.098	0.276	0.342	0.276	0.254	0.266	0.221	0.152	0.243	0.429	0.000	0.170	0.112	0.075	0.014	0.099	0.177	-0.002	0.074	0.099
**OH2**	0.286	0.368	0.304	0.393	0.544	0.340	0.214	0.078	0.114	0.256	0.317	0.338	0.370	0.113	0.482	0.388	0.000	0.061	0.158	0.219	0.142	0.231	0.114	0.169	0.147
**PA5C**	0.250	0.294	0.458	0.311	0.440	0.302	0.259	0.036	0.238	0.406	0.320	0.397	0.280	0.214	0.563	0.261	0.142	0.000	0.126	0.193	0.142	0.266	0.028	0.143	0.165
**MI1**	0.014	0.138	0.393	-0.082	0.487	0.060	0.114	0.337	0.075	0.322	0.304	0.349	0.105	0.297	0.453	0.181	0.339	0.267	0.000	0.048	0.094	0.222	-0.009	0.062	0.092
**MI2**	0.182	0.360	0.420	0.026	0.445	0.074	0.250	0.447	0.256	0.095	0.230	0.213	0.183	0.318	0.358	0.037	0.469	0.409	0.115	0.000	0.063	0.199	0.029	0.075	0.084
**OH1**	0.148	0.426	0.143	0.148	0.467	0.208	0.197	0.280	0.202	0.605	0.013	0.340	0.304	0.255	0.272	0.244	0.324	0.315	0.216	0.158	0.000	0.238	0.073	0.098	0.075
**FL1**	0.556	0.542	0.631	0.539	0.496	0.334	0.485	0.578	0.486	0.256	0.598	0.502	0.360	0.489	0.554	0.482	0.571	0.613	0.528	0.516	0.597	0.000	0.199	0.220	0.206
**NH1**	0.088	0.169	0.422	0.007	0.350	0.072	0.144	0.197	0.140	0.382	0.233	0.240	0.082	0.207	0.458	-0.006	0.263	0.069	-0.023	0.074	0.178	0.510	0.000	0.048	0.088
**MA1**	0.284	0.262	0.470	0.136	0.335	0.081	0.376	0.403	0.249	0.248	0.283	0.449	0.319	0.339	0.509	0.187	0.376	0.316	0.145	0.185	0.235	0.558	0.120	0.000	0.187
**VT1**	0.024	0.448	0.224	0.218	0.609	0.274	0.088	0.297	0.184	0.248	0.253	0.189	0.225	0.195	0.221	0.248	0.340	0.363	0.215	0.209	0.187	0.541	0.215	0.422	0.000
